# Vahagn: VisuAl Haptic Attention Gate Net for slip detection

**DOI:** 10.3389/fnbot.2024.1484751

**Published:** 2024-11-06

**Authors:** Jinlin Wang, Yulong Ji, Hongyu Yang

**Affiliations:** ^1^National Key Laboratory of Fundamental Science on Synthetic Vision, Sichuan University, Chengdu, China; ^2^School of Aeronautics and Astronautics, Sichuan University, Chengdu, China

**Keywords:** multimodal perception, multimodal deep learning, attention mechanism, haptic, robot perception

## Abstract

**Introduction:**

Slip detection is crucial for achieving stable grasping and subsequent operational tasks. A grasp action is a continuous process that requires information from multiple sources. The success of a specific grasping maneuver is contingent upon the confluence of two factors: the spatial accuracy of the contact and the stability of the continuous process.

**Methods:**

In this paper, for the task of perceiving grasping results using visual-haptic information, we propose a new method for slip detection, which synergizes visual and haptic information from spatial-temporal dual dimensions. Specifically, the method takes as input a sequence of visual images from a first-person perspective and a sequence of haptic images from a gripper. Then, it extracts time-dependent features of the whole process and spatial features matching the importance of different parts with different attention mechanisms. Inspired by neurological studies, during the information fusion process, we adjusted temporal and spatial information from vision and haptic through a combination of two-step fusion and gate units.

**Results and discussion:**

To validate the effectiveness of method, we compared it with traditional CNN net and models with attention. It is anticipated that our method achieves a classification accuracy of 93.59%, which is higher than that of previous works. Attention visualization is further presented to support the validity.

## 1 Introduction

With the growing need for industrial and service robots, which need to perform a range of grasping and complex manipulation tasks, the field of robot manipulation has attracted global attention from researchers (Liu et al., 2023a; Fang et al., 2023). The enhancement of a robot's overall operational capability relies on the attainment of stable and dependable grasping capabilities. Consequently, the assessment of grasping outcomes represents a pivotal domain for investigation.

The extensive study of existing computer vision achievements has facilitated the thorough examination of vision-based environmental perception technology. Researchers have achieved a significant number of exemplary works in the areas of slip detection (Mahler et al., 2019; Sundermeyer et al., 2021), material recognition (Liu et al., 2023a; Holm et al., 2020), and so on (Piacenza et al., 2022). In scenes driven by visual perception, computer vision can only acquire a limited amount of information from a single viewpoint. This information mainly includes a complete scene description, which is often affected by exposure and focus problems caused by changes in light and materials. With these limitations, vision-only methods still perform insufficiently in unstructured scenes. In situations where lighting conditions are far from ideal (Yi et al., 2022), unexpected obstructions happen (Phelan et al., 2017), or complex interactions with the target are needed (Wang et al., 2021), indirect measurements like vision are likely to be inadequate, which often lead to task failures.

In contrast, haptic sensing is receiving increasing research attention due to its direct access to interacting information. Vision could provide a comprehensive overview, which acquires generalized sensing, while haptic offers detailed records of contact, haptic acquires detailed and localized information through direct contact, combining the benefits of vision and haptic for detection is a more competitive option. The integration of visual and haptic modalities has been demonstrated to enhance the perception of robots, with related methods exhibiting superior performance in material classification (Xiong et al., 2023b; Yang et al., 2022; Xiong et al., 2023a), object recognition (Xiong et al., 2023c; Tatiya and Sinapov, 2019) and environment exploration (Liao et al., 2024; Luo et al., 2017).

One advantage of utilizing multiple modalities is that the remaining modalities can compensate for and provide information from disparate perspectives, even when some of the other modalities appear to be occluded or noisy. In modal pairing and perceptual tasks, when disparate inputs are encountered, humans employ “causal inference” to ascertain whether the two sensory signals originate from a single source (Ernst and Banks, 2002; Landy et al., 2011). This enables them to select the optimal means of integrating the acquired information. This crucial mechanism in humans has not yet been extensively explored with regard to its potential applications in the domain of visual and haptic fusion.

Concurrently, the data within the unimodal state is frequently heterogeneous. The most common approach is to extract features according to temporal and spatial dimensions, respectively. With regard to space, objects exhibit diverse shapes and materials, which can give rise to notable variations in friction when grasping them in different positions. In the context of time, despite a grasping action being relatively brief, it is a continuous process. The continuity and stability of this process also exert a significant influence on the outcome of the grasping action.

In this paper, we focus on how to learn effective Visual-Haptic features in the task of slip detection. In our approach, we utilize data enhancement and preprocessing techniques to process sequential data comprising visual and haptic images. Next, we extracted temporal and spatial features using self-attention (Vaswani et al., 2017) and Squeeze-and-Excitation attention (Hu et al., 2018), respectively. In the fusion stage, two-step fusion is employed to achieve the integration of visual and haptic features, as well as the fusion of temporal and spatial features, respectively. In each step, gate units are employed to regulate the input signal. After that, fusion feature is sent to a MLP to get the prediction. We validate the performance of the model and compare it with several state-of-the-art (SOTA) methods on slip datasets (Li et al., 2018). The experiment results demonstrate that our method exhibits superior performance compared to state-of-the-art (SOTA) methods. The visualization of the attention mechanism illustrates that our method is capable of effectively extracting both temporal and spatial feature from visual and tactile information.

The main contributions of the work in this paper can be described as follows:

1. We propose a feature extraction method that combines two attention mechanisms to extract features in spatial and temporal dimensions, improving the model's ability to perceive spatial-temporal feature;
2. Balance and causal inference mechanisms in neurological research are considered in our method, improving the model's ability to adapt to multimodal inputs;
3. A two-step fusion model was employed to enhance the information fusion capability of the method;
4. The experiment results show that our method is superior to other three SOTA methods in terms of the accuracy with 5%;
5. Analysis of the visualization suggest that the Vahagn model can capture position-related features and time-related features that are useful for task.

## 2 Realated work

### 2.1 Haptic sensors

A number of haptic sensors have been developed in recent years. Some sensors are designed to capture forces and moments on contact, or data from other sensors such as temperature and vibration directly. BioTac (Wettels et al., 2014) is an excellent example. The sensor has a rigid core and several types of sensors inside, surrounded by a deformable housing. The high-resolution haptic sensors represented by GelSight (Dong et al., 2017) are quite different, as the surface of the sensor is an elastic gel that changes according to the shape of the object in contact. A normal tiny camera is placed under the elastomer, and in order to make the shape changes observed more visible, three LEDs are placed in different directions around the gel. Thanks to the unique design, GelSight could detects three different types of information: movement of the object texture, change of the contact area, and stretching of the sensor surface. An important advantage of using cameras to capture signals is that the data is standardized as images, which allows researchers to use well-established computer vision algorithms to extract the features.

### 2.2 Slip detection

Slip detection is very important for robotic manipulation. In recent years, various methods have been employed. Liao et al. (2024) attempted to use graph networks to evaluate grasping stability on pressure data obtained from a novel PT-TIP sensor. Liu et al. (2023a) used a triple network to judge the grasping result with haptic images, and they found that haptic can still achieve similar results under different grasping postures. The results demonstrate the importance of combining visual and haptic information for perceiving joint tasks. Li et al. (2018) proposed a DNN based on CNN and LSTM. The model uses pre-trained CNN to extract features from images of vision and haptic. Multiple sets of spliced features are then sent into LSTM (Hochreiter and Schmidhuber, 1997) for fusion, and the grasping stability results are outputted. The experiments result proved the effectiveness of the joint perception of visual and haptic (Wang et al., 2022; Zhou et al., 2023; Girbes-Juan et al., 2020). Inspired by the success of attention mechanisms on dependencies between images (Zhao et al., 2020; Zhu et al., 2023), words (Yang et al., 2021; Munkhdalai et al., 2024), and audios (Ryan et al., 2023), (Cui et al., 2020) introduced the attention mechanism to the feature fusion process. They proposed the VTFSA model, which uses the self-attention mechanism on the spliced features after extracting the input visual and haptic separately. The comparison results proved its effectiveness. However, this processing does not effectively distinguish the importance of the information from the two sources and underutilizes the information from the time series. Han et al. (2023) introduced a Transformer-based robotic grasping framework for rigid gripper robots, leveraging haptic and visual information to ensure secure object grasping. Evaluation demonstrated that Transformer models exhibit higher grasping accuracy and computational efficiency compared to traditional CNN-LSTM models. Although the methods proposed by these works can reach a decent performance on slip detection, none of them have taken into account the problem of adaptation between visual-haptic information and the spatial-temporal feature of the grasping process in a unified way. Therefore, we proposed a new method involves performing temporal and spatial feature extraction on input visual and haptic sequences in parallel, and get the feature of multi-source and spatial-temporal information with a two-step fusion.

### 2.3 Attention mechanisms

In recent years, the attention has been widely used in various tasks, including natural language processing (Yang et al., 2021; Munkhdalai et al., 2024), image feature recognition (Zhao et al., 2020; Zhu et al., 2023), and so on (Yak et al., 2023; McKinzie et al., 2024). The self-attention (SA) (Vaswani et al., 2017) has been formalized to better capture the interrelationships between input words, resulting in a significant improvement in machine translation performance. To better account for the varying importance of individual channels in image features, Hu et al. (2018) proposed the Squeeze-and-Excitation attention (SEA) mechanism. This mechanism allows the network to focus on specific feature channels by assigning different weights of importance to them or by suppressing channel features that are not useful enough. Here, we adopt ideas from SA mechanism and SEA mechanism into the spatial and temporal feature extraction modules, respectively, so that key spatial locations can be extracted and interrelationships between time series can be recognized.

## 3 Methods

In Section 3.1, we introduce the dataset used here in detail. In Section 3.2, we provide a complete description of Vahagn.

### 3.1 Slip dataset

Li et al. (2018) created a multimodal dataset that comprises visual and haptic data. They collected 1102 sets of interaction data from 84 different common objects. The collection experiment employed a UR5 robotic arm equipped with a WSG-50 parallel gripper. To obtain haptic data, a GelSight sensor was added to one side of the gripper. In experiment, a camera was added to the middle of the gripper jaws to capture visual observation. During each execution, the position and strength of the grasp varied, and the data from the camera and haptic sensors were recorded simultaneously. Unlike the Calandra dataset (Calandra et al., 2017), which only collected data at several key moments, Li et al. (2018) collected sequences data of visual and haptic throughout the execution. The results of each grasping were recorded as labels. In the Slip dataset, each set of data comprises two image sequences captured by the camera and the haptic sensor, both with a resolution of 640 × 480. The visual and haptic images are captured simultaneously and are both 21 frames in length. A label is provided for each set, denoting the result of the grip. The value “0” signifies a sliding movement, whereas “1” denotes a stabilizing action.

### 3.2 The overall framework

**Figure 1:**
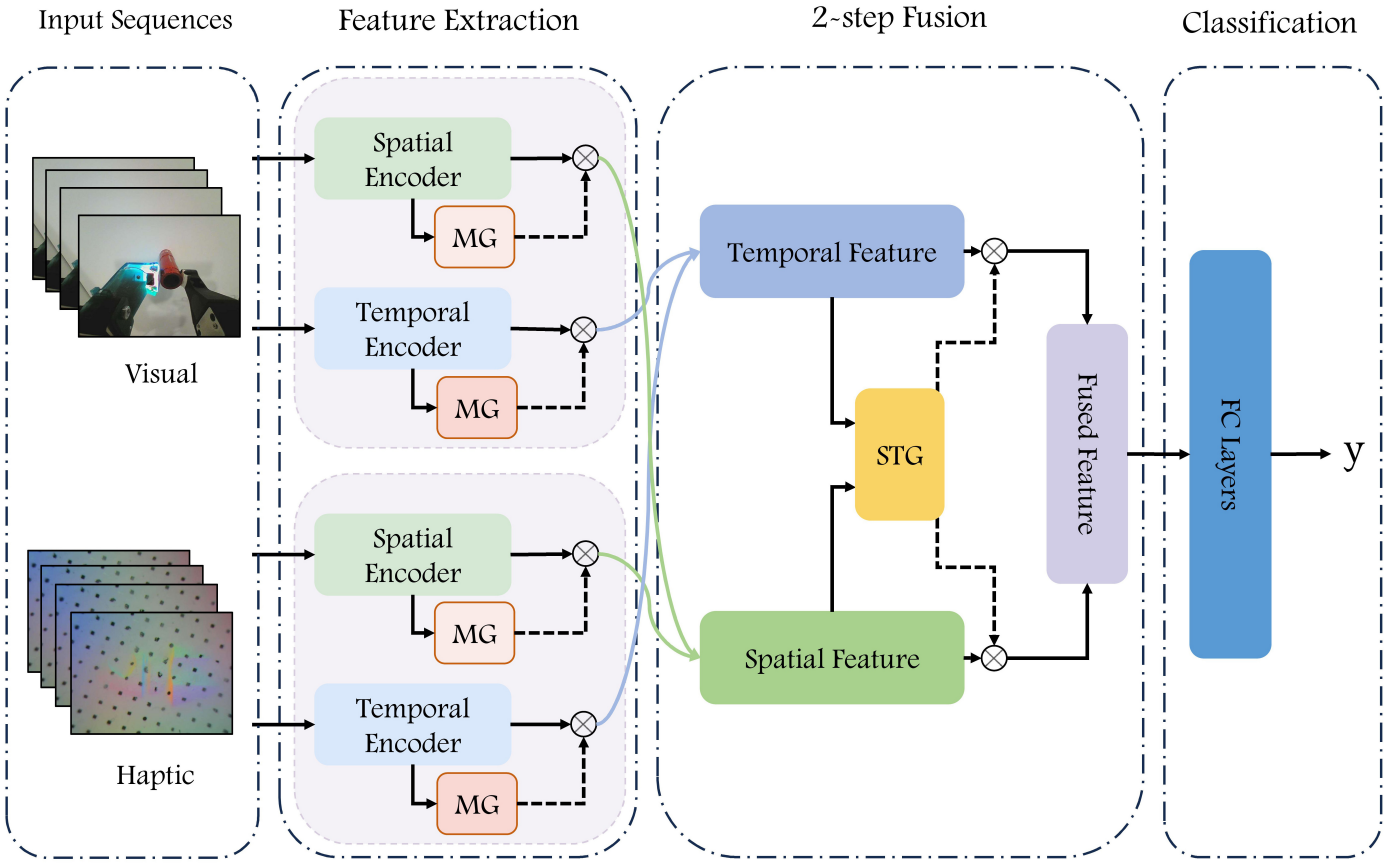
Diagram of our VisuAl Haptic Attention Gate Net (Vahagn) model. The RGB images and the haptic sensors images are fed into a deep neural network to predict whether the grasp is successful. Consecutive *N* frames of visual and haptic images are inputted into the temporal encoder and spatial encoder, respectively, after which four sets of features from vision and haptic are fused twice to obtain the resulting features, which are concatenated as the input into a fully connected network for prediction.

The model proposed in this paper takes visual and haptic image sequences as inputs and extracts temporal and spatial features using self-attention (SA) and Squeeze-and-Excitation attention (SEA), respectively. In the fusion process, the weights of different features are adjusted with the help of modality gate unit and temporal-spatial gate unit. The attention-based feature extraction module for visual-haptic inputs and the gate-based feature fusion module together constitute the VisuAl Haptic Attention Gate Net (Vahagn), and the overall framework of the model is shown in Figure 1.

The proposed network has four main components: data processing, feature extraction, two-step fusion, and classification modules.

#### 3.2.1 Data processing

**Figure 2:**
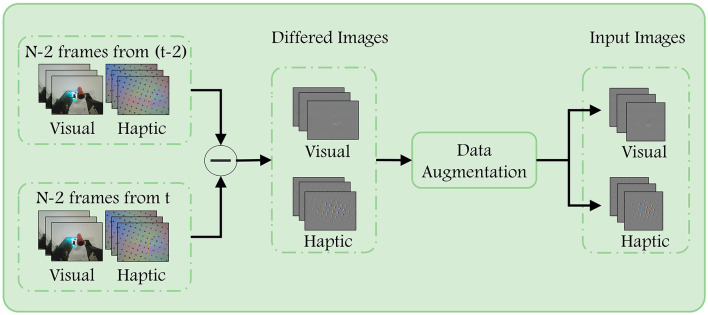
Data preprocessing. First, we subtract the corresponding two frames ago from each of the input images to highlight the regions that have changed due to contact with the target object. After that, we flip the images of the same group using the same probability after resizing to complete the enhancement of the training data.

The stability of the entire grasp is contingent upon the stability of all its constituent segments. Therefore, we propose that the label assigned to a given grasp set can also be applied to the its sub-segments. In accordance with this supposition, we employ *N* frames of successive visual and haptic images as inputs for a single prediction, here we set *N* to 8. The Figure 2 illustrates the processing and enhancement performed prior to the input being passed to the network. .

To emphasize the difference between the current frame image and the previous ones, we first subtract the images across frames. The *N*−2 images starting from *t* are differenced from the corresponding images starting from *t*−2. This helps to eliminate task-irrelevant background in the images that is not necessary for completing the task. We apply the same operation to both the visual images and haptic images. Afterwards, in order to increase the diversity of the data, the image was resized to 224 × 224, and a decision was made as to whether to flip it horizontally or vertically, with a probability of 0.5. The operations performed are consistent across the visual difference image sequence and the haptic difference image sequence, respectively.

#### 3.2.2 Feature extraction

When performing grasping, the selection of grasping position and the continuity of the action during execution have a great influence on the success of grasping. In this paper, with visual image sequence $X_{v}\in R^{\left(N-2\right)\times H_{v}\times W_{v}}$ and haptic image sequence $X_{h}\in R^{\left(N-2\right)\times H_{h}\times W_{h}}$ as inputs, we constructe a spatial encoder and a temporal encoder, for extracting spatial feature about grasping position and temporal feature about stability information. Here, *N*, *H*, and *W* denote the sequence length, image height, and image width. Following data processing, both H and W yielded a value of 224.

##### 3.2.2.1 Spatial encoder

**Figure 3:**
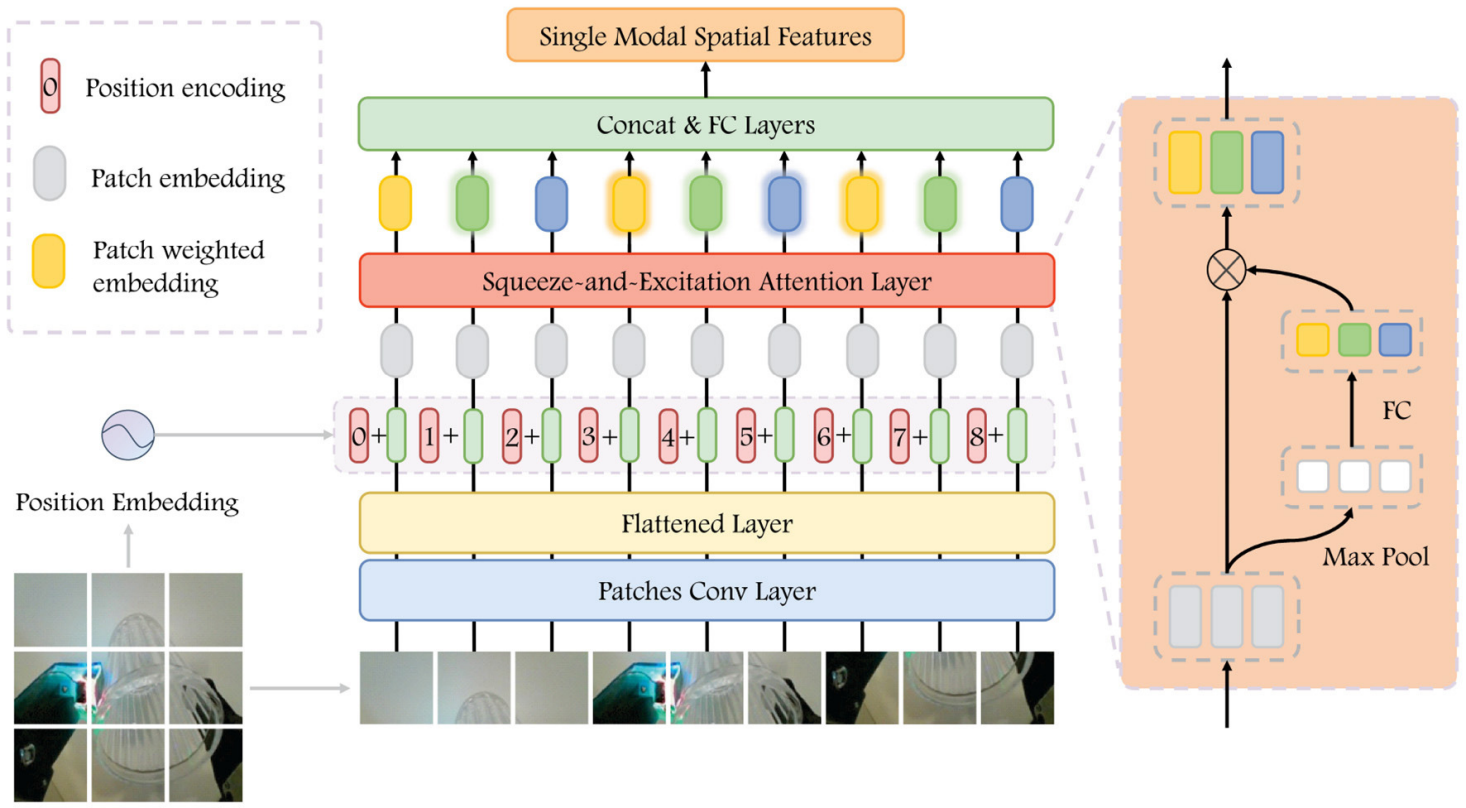
Visualization of Spatial Encoder. The input image is patched to embedding, and we get the patch embedding after adding the space position embedding. After passing it through the SEA, we have weighted features. The single-modal spatial features are obtained after concatenated them as the input into a fully connected network.

The purpose of the spatial encoder is to identify and extract features associated with the spatial position of the grasping. From the perspective of visual analysis, our focus is on the relative position of the gripper and the object, as well as the posture of target. In contrast, from the tactile perspective, we place emphasis on the specific details of the contact between the gripper and the target. The structure devised for the spatial encoder is illustrated in Figure 3. The encoder takes a single image as input and outputs the single modal spatial features.

In the visual difference image sequence, the final frame is selected to the spatial encoder as input and extracted the patches $X_{pV}^{S}\in R^{p_{vn}^{S}\times p_{vh}^{S}\times p_{vw}^{S}}$, where $p_{vh}^{S}\times p_{vw}^{S}$ is the resolutions of each patch and $p_{vn}^{S}=\frac{H_{v}\times W_{v}}{p_{vh}^{S}\times p_{vw}^{S}}$ is the number of patches, which we set to 64. Subsequently, the patches go through a convolutional layer to extract relevant feature. Then, we flatten it and added position embedding to get the patches feature $F\in R^{p_{vn}^{S}\times D^{S}}$, *D*^*S*^ denotes the feature length of each patch and takes the value 768. In feature *F*, each row represents the feature of one patch, which contains information regarding the position within the original image. We then apply the Squeeze-and-Excitation attention (SEA) mechanism on *F* to extract the relative importance of individual patch. The execution process can be represented as:


(1) $$\begin{array}{l} F^{\prime }=F⊙Sig\left(MLP\left(Pool\left(F\right)\right)\right), \end{array}$$

Feature extraction is conducted through the maximum pooling layer and FCN, and then sigmoid activation is applied to obtain importance weights. After that, these importance weights are then multiplied with the original input *F* to generate the weighted representation of patches feature.

For the haptic sequence, we also use the last frame of the difference image as input and perform the same process. The spatial encoder for haptic utilizes the identical hyperparameters as that for vision, with their parameters updated independently. The spatial encoder provides us with visual spatial features $F_{V}^{S}$ and haptic spatial features $F_{H}^{S}$.

##### 3.2.2.2 Temporal encoder

The temporal encoder takes a series of difference images as input to obtain continuity and stability during execution. The input visual difference image sequence is denoted as $X_{pV}^{T}\in R^{\left(N-2\right)\times p_{vh}^{T}\times p_{vw}^{T}}$, where $p_{vh}^{T}\times p_{vw}^{T}$ is the resolution of input images. We use a convolutional layer to extract the 2D features, flatten and add them with the time encoding to obtain the temporal feature *V*^*T*^∈*R*^(*N*−2) × *D*^^*T*^, where *D*^*T*^ is the feature length of single-frame and is set to 512.

To extract the relationships between frames in the time dimension, a self-attention unit is introduced, inspired by works about natural language processing. The self-attention mechanism can be represented as the process of matching a query (*Q*) and a set of key (*K*)-value (*V*) pairs to an output. In execution, the *Q*, *K*, and *V* matrices are generated by multiplying the input vector with three separate learnable matrices *W*^*Q*^, *W*^*K*^, and *W*^*V*^, respectively:


(2) $$\begin{array}{l} Q=XW^{Q},K=XW^{K},V=XW^{V}, \end{array}$$

where the vector lengths for *Q*, *K*, and *V* are all set to 128.

The attention scores for different positions are obtained by computing the dot product of *Q* and *K*. These scores are then scaled and multiplied with the matrix *V* to extract a single attention, which is denoted as *Attention*(*Q, K, V*). The attention scores could capture the significant relationship between the frames in a continuous sequence. Then the weighted features obtained by multiplying the attention scores with the original inputs are used as the temporal feature, and we denote the temporal features from vision and haptic as $F_{V}^{T}$ and $F_{H}^{T}$. By focusing on the correlation between the difference images at different locations in the input sequence, one can gain insight into the variability between these images. In turn, such insight facilitates the assessment of the overall process continuity and stability.

#### 3.2.3 Two-step fusion

**Figure 4:**
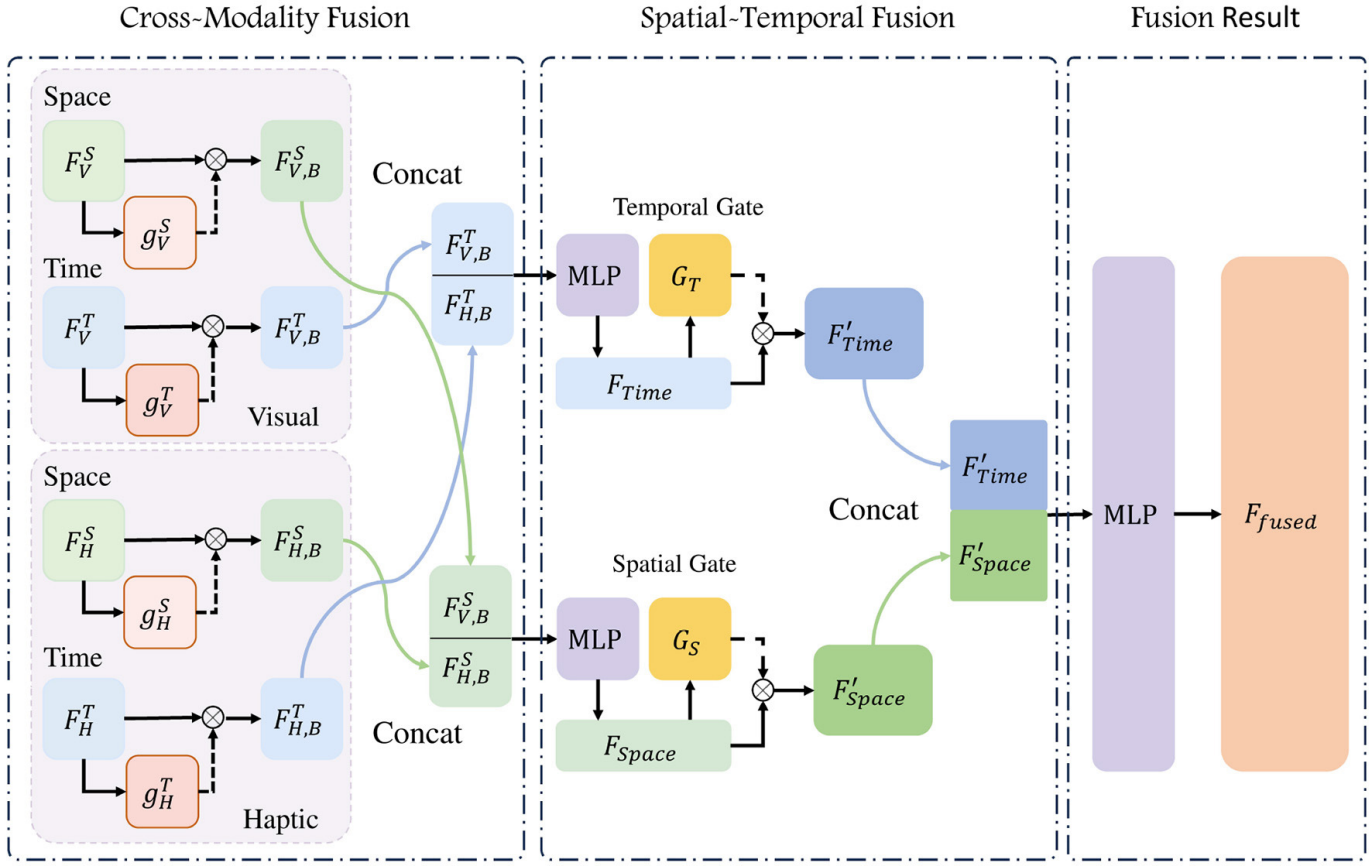
Flowchart of Two-step Fusion. The temporal and spatial features derived from both vision and haptics are integrated through a two-step process: cross-modality fusion and spatial-temporal fusion. In this process, two gates—one for modal fusion and another for spatio-temporal fusion—are employed to regulate the relative weights of the features.

Spatial feature has the information of the location of contact and the posture of the target, while temporal feature focuses on the continuity and stability of the whole process. The input data are derived from both visual and haptic sources, and after feature extraction by the temporal and spatial encoders, we can get a total of four different sets of features. To fully exploit the cross-modal information from both time and space, we employ a two-step feature fusion, which includes Cross-Modality fusion and Spatial-Temporal fusion, as illustrateed in Figure 4, and get a joint feature of the inputs images.

##### 3.2.3.1 Cross-modality fusion

We fused spatial features from vision and haptic, as well as the temporal features from the same sources. Given that the data utilized in this model is derived from both visual and haptic modalities, this fusion process is called Cross-Modality fusion. From haptic images, we can observe the localized pressing situation, but the perception of information such as the gesture of the target is lacking, while the information from vision is more comprehensive, but deficient in detail. When comes to how much each modality could help the prediction, we look at the neurological study for inspiration. Rideaux et al. (2021) mentioned the balance mechanisms and causal inferences processes when they receive information from different sources. Based on this, we put the original features *F* from visual and haptic into modality gate (MG) units for importance scores *g*(*F*), which in turn is multiplied with the original features *F* for balanced features *F*_*B*_. The gate unit employed in this study is a three-layer fully connected layer, wherein the input length is identical to the output length and is equal to the length of the feature *F*. The processing of the gate unit can be represented as:


(3) $$\begin{array}{l} F_{B}=F⊙g\left(F\right). \end{array}$$

We then concatenate the weighted features and pass them to a FCN and get the temporal feature *F*_*Time*_ and spatial feature *F*_*Space*_ with length 1, 024. These two can be expressed as:


(4) $$\begin{array}{l} F_{V,B}^{S}=F_{V}^{S}⊙g_{V}^{S}\left(F_{V}^{S}\right), \end{array}$$

(5) $$\begin{array}{l} F_{H,B}^{S}=F_{H}^{S}⊙g_{H}^{S}\left(F_{H}^{S}\right), \end{array}$$

(6) $$\begin{array}{l} F_{Space}=MLP\left(Concat\left(F_{V,B}^{S},F_{H,B}^{S}\right)\right); \end{array}$$

(7) $$\begin{array}{l} F_{V,B}^{T}=F_{V}^{T}⊙g_{V}^{T}\left(F_{V}^{T}\right), \end{array}$$

(8) $$\begin{array}{l} F_{H,B}^{T}=F_{H}^{T}⊙g_{H}^{T}\left(F_{H}^{T}\right), \end{array}$$

(9) $$\begin{array}{l} F_{Time}=MLP\left(Concat\left(F_{V,B}^{T},F_{H,B}^{T}\right)\right), \end{array}$$

where we use superscripts to distinguish the encoder, and subscripts to indicate the source of the data, in detail, the superscripts *S* for spatial encoder and *T* for temporal encoder, while the subscripts *V* and *H* denote features from vision and haptic. The *g* denotes the modality gate applied on the modality features. The effectiveness of the modality gate will be fully validated in subsequent ablation experiments.

##### 3.2.3.2 Spatial-Temporal fusion

Spatial features derived from visual and haptic inputs are integrated in *F*_*Space*_, and temporal features derived from visual and haptic inputs are integrated in *F*_*Time*_. When temporal and spatial features are fused, a similar three-layer FCN is employed to construct a gated unit *G*, which serves to adjust the relative weights of temporal and spatial features. In detail, we pass *F*_*Time*_ and *F*_*Space*_ through separate spatial-temporal gate *G* for weighted feature $F_{Time}^{\prime }$ and $F_{Space}^{\prime }$. Gate unit *G* does not change the length of the features. We then concatenate the two feature vectors and mapping it to the fused feature of length 512 use a fully connected network:


(10) $$\begin{array}{l} F_{Time}^{\prime }=F_{Time}⊙G\left(F_{Time}\right), \end{array}$$

(11) $$\begin{array}{l} F_{Space}^{\prime }=F_{Space}⊙G\left(F_{Space}\right), \end{array}$$

(12) $$\begin{array}{l} F_{fused}=Concat\left(F_{Time}^{\prime },F_{Space}^{\prime }\right)W^{T}+b, \end{array}$$

where *F*_*fused*_ represents the complete fused feature of the current input set. *W*^*T*^ is a learnable parameter that performs linear mapping, and *b* represents another set of learnable parameters used to provide offsets.

#### 3.2.4 Classification

The objective of this task is to predict the stability of grasping, indicated by a binary output of either 1 for stable grasping or 0 for slipping. We pass the fused feature *F*_*fused*_ to a three-layer FCN to get a two-dimensional output, which represents the predicted probability at the corresponding label.

## 4 Experiment

In this section, in order to verify the validity of the model proposed, we conduct experiments on the dataset proposed in Li et al. (2018). We take haptic and visual images sequences as input, and predict the state of grasping with the output. We compare it with three state-of-the-art methods, CNN-LSTM (Li et al., 2018), VTFSA (Cui et al., 2020), and the TimeSformer (Han et al., 2023). In addition, we conducted a series of ablation experiments and analyzed the results to understand the role of each component. For the encoders, we visualized the attention and analyzed the features obtained from the inputs.

### 4.1 Implementation details

We conduct the experiments on the slip dataset. In the experiments, the dataset is divided into training, validation, and test sets. During training, we increase the diversity of the data with augmentation using the methods described previously. The results were obtained on the test dataset. Three different inputs were tested for each model: vision alone, haptic alone, and vision with haptic.

For the CNN-LSTM model, we follow Li et al. (2018) and choose VGG16 as the backbone of the CNN with pre-trained initialization. After extracting features from visual and tactile images respectively using pre-trained CNN model, the features are concatenated and passed into the LSTM, and then the out features are sent to a FCN to get the prediction. For VTFSA, we follow the description provided by Cui et al. (2020). The input images and haptic signals are processed in discrete encoders to obtain the visual feature, and the haptic feature. These features are then conveyed to the self-attention module after concatenation. Finally, the fused features are subsequently transmitted to the downstream FCN classifier, where the prediction is generated. In regard to TimeSformer, we follow Han et al. (2023). The temporal and spatial features of the visual and haptic sequences are extracted in a sequential manner, using a space-time attention method. The visual and haptic features are then concatenated and passed directly to the downstream prediction FCN model.

In experiments, we took 8-frame vision images and 8-frame haptic images as input. The predicted probabilities and labels were provided to the cross-entropy function to get the loss values. The Adam optimizer was utilized in conjunction with a learning rate of 8 × 10^−4^. A total of 200 rounds were conducted in batches of 48. The comparison results are displayed in the Table 1.

**Table 1:** Performance comparison of different models with different inputs on slip dataset.

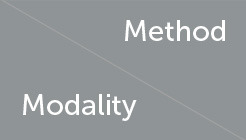	**CNN-LSTM (VGG16)**	**VTFSA**	**Time Sformer**	**Vahagn**
Vision-only	55.13%	–	78.65%	81.02%
Haptic-only	81.84%	–	81.04%	84.72%
Vision & Haptic	87.76%	88.46%	87.88%	**93.59%**

Vahagn outperforms the traditional CNN-LSTM method and attention-based methods VTFSA and TimeSformer. Bold values indicate the optimal results.

### 4.2 Evaluation

From Table 1, we can see that the Vahagn can provide classification results with higher accuracy. For the CNN-LSTM, haptic-only with 81.84% significantly outperforms vision-only with 55.13%, as shown by Liu et al. (2023b), and Transformer models perform similarly for single modality case. For multi-modalities case, Vahagn achieves 93.59% with accuracy, which is +5% higher than VTFSA (88.46%) and +5% higher than TimeSformer (87.88%), similar result was obtained when compared to traditional CNN-LSTM method. Vahagn has better performance than traditional methods and other Transformer models in the slip detection task.

To understand the importance of temporal and spatial information in prediction, we conducted ablation experiments on encoder. The results on accuracy, precision, and recall are shown in Table 2. From the results, we can see that, the accuracy is acceptable for spatial-encoder-only with 81.31%, but the model struggles to provide effective results when spatial encoder is missing. The accuracy is significantly better when the input is jointly encoded with temporal encoder and spatial encoder (93.59%).

**Table 2:** Ablation studies results of Vahagn on encoder.

**Temporal encoder**	**Spatial encoder**	**Acc**	**Pre**	**Recall**
w/	w/	**93.59%**	**91.13%**	**97.44%**
w/	w/o	51.52%	50.77%	91.92%
w/o	w/	81.31%	81.00%	81.82%

The incorporation of temporal encoder and spatial encoder has been demonstrated to result in a 12.28 and 42.07% improvement in accuracy, respectively, the simultaneous use of both encoders has been shown to significantly enhance the Vahagn. Bold values indicate the optimal results.

The results show that Vahagn not only outperforms the traditional method of CNN-LSTM, but also compares more favorably with VTFSA, which also adopts the attention mechanism. And Vahagn performs equally well against the TimeSformer, which takes into account the spatial-temporal features.

In comparison with the CNN-LSTM, the Vahagn employs a distinctive design for the extraction of temporal and spatial features. In contrast to VTFSA, Vahagn considered the relative importance between different source and used a gate unit to regulate it, enabling Vahagn to adjust the weight of visual and haptic information. In contrast to the TimeSformer, we distinguished between temporal and spatial feature extraction and the two processes are relatively independent, which helps to preserve deeper features from the input. Furthermore, the ablation experiment on encoder validates the assumption that the input image sequences contain different information about time and space.

In order to validate the veracity of Vahagn's structure and key parameters, we conduct experiments on the length of sequence input into the temporal encoder *N* and the size of the patches in the spatial encoder. We also perform ablation experiments on several key structural parts of Vahagn.

We firstly test on the length of input sequence *N*. Table 3 displays the results as the input sequence length *N* variations. We train the Vahagn with different input lengths (6, 7, 8, 9, and 10) and collected the corresponding accuracy, precision, and recall results. The results show that the best accuracy was obtained when *N* is 8, accompanied by the best pre and recall, while 10-frame input also showed strong competitiveness.

**Table 3:** Performance comparison of Vahagn, with different input length *N*.

***N***	**Acc**	**Pre**	**Recall**
6	88.89%	85.32%	93.93%
7	90.08%	85.32%	96.83%
8	**93.59%**	**91.13%**	**97.44%**
9	90.28%	87.18%	94.44%
10	91.41%	87.96%	95.96%

The Vahagn model performs best when *N* is 8. Bold values indicate the optimal results.

When comes to the patch size *p*_*h*_×*p*_*w*_ of spatial encoder, we selected two parameter sets with patch sizes 28 × 28 and 56 × 56 for comparison. The results are listed in Table 4. From the result, we can see that smaller patch lead to better classification accuracy, with about 5% beter.

**Table 4:** Performance comparison of Vahagn, with different patch size.

**Patch size**	**Acc**	**Pre**	**Recall**
28 × 28	**93.59%**	**91.13%**	**97.44%**
56 × 56	90.60%	89.26%	92.31%

Smaller patch size result in higher accuracy performance. Bold values indicate the optimal results.

After that, we also need to verify the necessity of the modality gate (MG) unit and spatial-temporal gate (STG) unit. We make experiments to compare the performance with MG missing, STG missing, and with both missing, respectively. The results of the comparison experiments are shown in Table 5. From this, we can see that both MG unit and STG unit has positive effects on improving the model's prediction accuracy. When both gate units are missing, Vahagn struggles to provide valid results for prediction.

**Table 5:** Ablation studies results on gate units (MG & STG).

**MG**	**STG**	**Acc**	**Pre**
w/	w/	**93.59%**	**91.13%**
w/o	w/	88.89%	85.83%
w/	w/o	90.17%	84.06%
w/o	w/o	60.49%	60.56%

Both MG and STG unit lead to performance improvements of 28 and 30%, respectively. Bold values indicate the optimal results.

To further validate the effectiveness of the attention used in encoders, we conducted the following comparative experiments:

**Vahagn + SEA + SA**: We use the SEA mechanism in the spatial encoder to extract key regions in the visual image and contact regions in the haptic; and the SA mechanism is used to extract correlations between frames in temporal encoder.

**Vahagn + SEA**: The same SEA as **Vahagn + SEA + SA** is used in spatial encoder; in temporal encoder, SEA module is used to replace SA.

**Vahagn + SA**: The same SA as **Vahagn + SEA + SA** is used in temporal encoder; in spatial encoder, SA module is used to replace SEA.

Despite employing different attentional mechanisms, the three experiments used consistent data preprocessing and parameter settings, We show the results of the accuracy, precision, and recall in Table 6.

**Table 6:** Comparative studies results of Vahagn, on attention.

	**Spatial attention**	**Temporal attention**	**ACC**	**Pre**	**Recall**
Vahagn + SEA + SA	SEA	SA	**93.59%**	**91.13%**	**97.44%**
Vahagn + SEA	SEA	SEA	91.88%	90.83%	93.16%
Vahagn + SA	SA	SA	49.79%	–	–

The method employing both SA and SCA demonstrated superior performance. Bold values indicate the optimal results.

As shown in Table 6, compared with Vahagn + SEA + SA, the accuracy decreases by 1+% after replacing the SA in the temporal encoder with SEA. And when the SEA of spatial encoder is replaced with SA, its accuracy came to 49%, which is pretty worse than the other. The results of the comparative experiments show that the joint use of the two attention mechanisms is more effective in helping Vahagn to extract the spatial-temporal feature in the sequences.

### 4.3 Attention analysis

To clearly explore why the Vahagn model has better performance, we visualize the scores calculated by the attention and analyze them with the input images. This approach allows for an intuitive understanding of the role played by input images in the feature extraction process.

**Figure 5:**
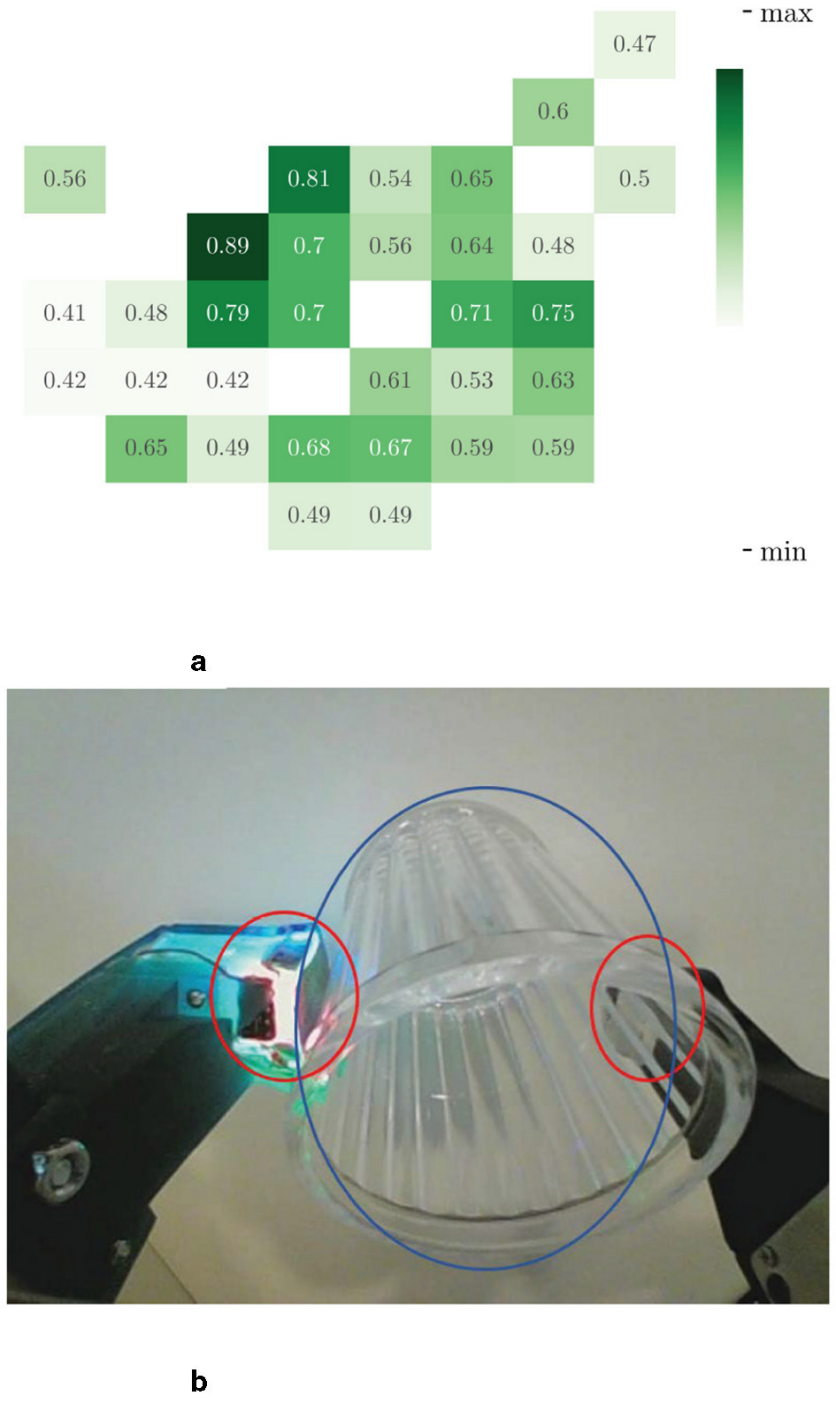
Visualization of spatial attention on the last frame of a visual input sequence. We show here the results for a set of three images. Figure **(a)** is the distribution of attention on the different patches and figure **(b)** is the original vision image. The image is split into 8 × 8 patches with size 28 × 28. The positions in figure **(b)** circled in red are the main contact points of the jaws for grasping, which received high scores in attention; the location circled in blue is the target, whose gesture is rendered by the attention distributed around the object contours. **(a)** SCA visualization. **(b)** Input vision.

Figure 5 shows the attention heat map visualization of visual input. The location of gripper-target contact in vision receives significant attention, which is circled in red in Figure 5b. Additionally, the object's occupied position received an above-average score, which is marked with a blue circle. This is crucial for effectively understanding the overall spatial information.

**Figure 6:**
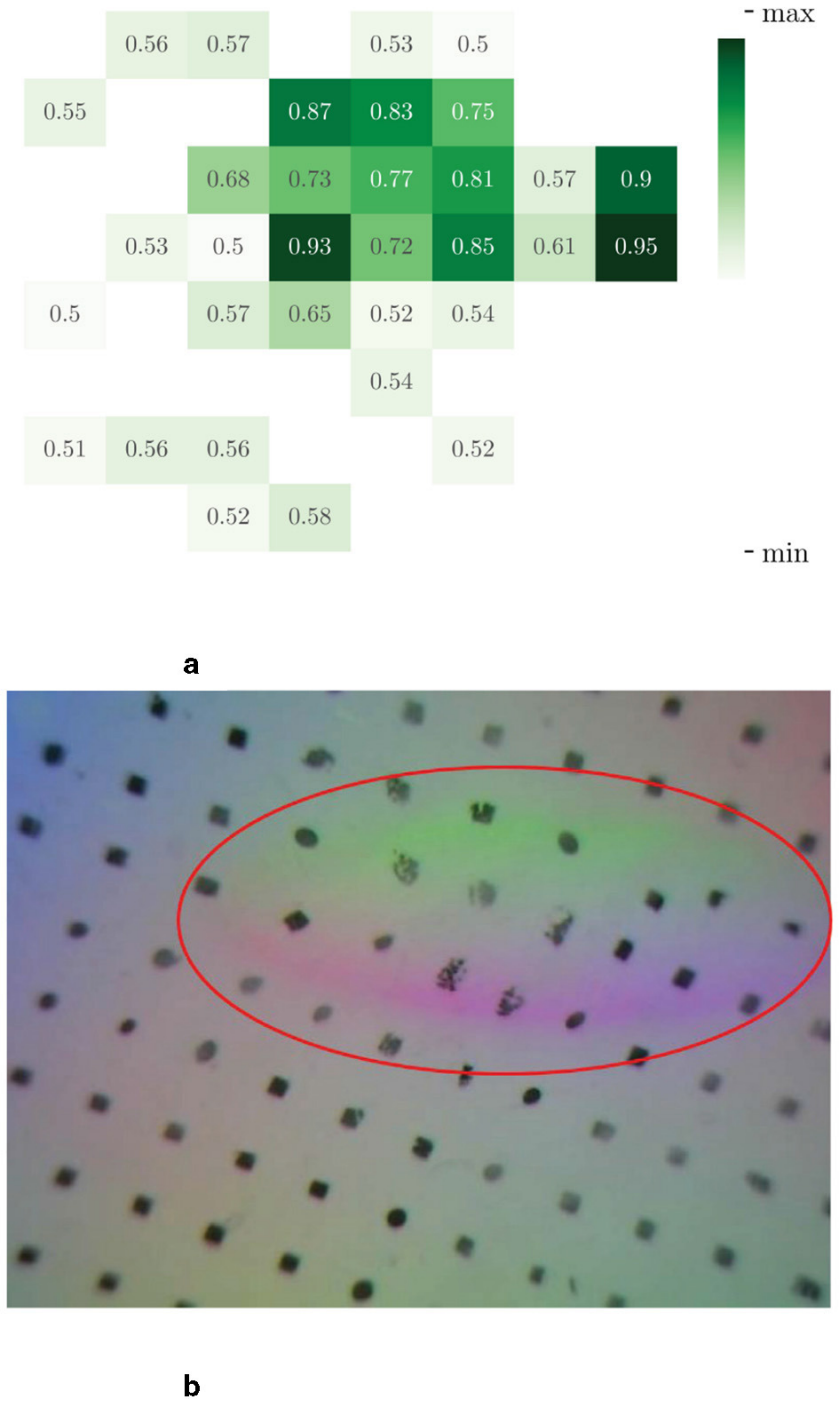
Spatial attention visualization of the last frame of a haptic input sequence. We show here the results for a set of three images. Figure **(a)** is the distribution of attention on different patches and Figure **(b)** is the haptic original image. The image is split into 8 × 8 patches with size 28 × 28. The position in Figure **(b)** circled in red is the traces acquired by the haptic sensors on the gel when the grasping occurs, and this region scored high in attention map. **(a)** SCA visualization. **(b)** Input vision.

Similarly, we visualized the attentional heat map for the haptic, as shown in Figure 6, where attention is mainly focused on the location of the contact (circled in red in Figure 5b). From the visualization result of spatial attention, we can see that spatial encoder could effectually extract the information about the contact position and the posture of target, which are critical for a successful grasping action.

**Figure 7:**
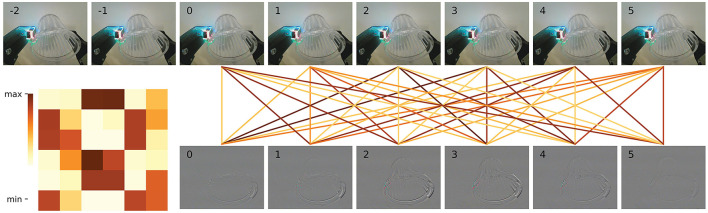
Temporal attention visualization of a single frame of input vision images to others in the sequence. The eight frames of the original image before data processing are shown at the top, and the images after data processing (without resizing) are shown at the corresponding position at the bottom. The attention maps between the six frames, which are input to the temporal encoder, are shown in the lower left.

**Figure 8:**
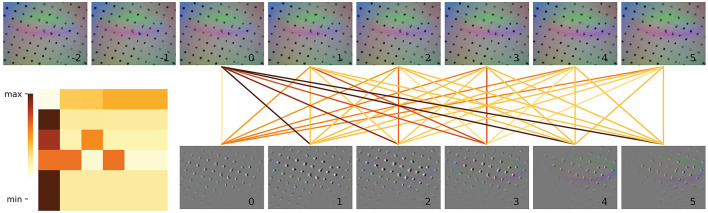
Temporal attention visualization of a single frame of input haptic images to others in the sequence. The eight frames of the original image before data processing are shown at the top, and the images after data processing (without resizing) are shown at the corresponding position at the bottom. The attention maps between the six frames, which are input to the temporal encoder, are shown in the lower left.

To more effectively demonstrate the relationship between the data at each time point, we utilize a visual representation of the correlation weights obtained through the temporal attention mechanism. The strength of the correlation is indicated by the darkness of the color of the connecting lines. Figure 7 shows the temporal correlation of the visual input images, with a heat map of the attention values located in the bottom left corner of the figure. The eight images bearing serial numbers from −2 to 5 at the top are the visual image inputs necessary to perform a single detection. The six images that are actually fed into the model are displayed below, indexed from 0 to 5. In comparison to the remaining four images, which primarily depict the outline of the glass mouth, the images with serial numbers 2 and 3 demonstrate a markedly enhanced delineation of the glass outline and correspond to considerably elevated attention values. It can be observed that the network assigns a higher degree of attention to images that exhibit discernible contrasts. This allows the model to capture visual variability in the input segments, thus enabling an assessment of the visual stability and continuity of the input. Similarly, the correlation between the sequence of haptic results is illustrated in Figure 8. The image with serial number 0 is indicative of the result of temporary instability of contact, and all subsequent images demonstrate a higher correlation with the image with serial number 0. The model demonstrates a heightened focus on haptic variation throughout the grasping process. This enables the model to observe the relative motion of the fingertips during execution, thereby facilitating an assessment of haptic stability and continuity.

### 4.4 Analysis

When examining the reasons for effectiveness, we consider both its network structure and method of feature extraction. One of the potential reasons is that we extract features from visual and haptic separately, and we dynamically adjust the importance share of visual and haptic by MG units, which is missing in the CNN-LSTM. Unlike the VTFSA, which connects the two features and applies the self-attention directly, it doesn't actually distinguish between information coming from the visual or haptic, and the importance of different sources is not captured. The two-step fusion method with gate units in Vahagn effectively facilitates two dynamic adjustments of the input features. Firstly, in the fusion of modal, adjusting the weight of the importance of visual and haptic information helps Vahagn to discard the noise that is difficult to bring effective discriminative information. Secondly, in the fusion of spatial-temporal feature, the incorporation of gate units into the fusion of spatial and temporal features enables the model to strike a more optimal balance between temporal stability assessment and spatial location accuracy assessment. This provides a more comprehensive and balanced discriminative basis for Vahagn.

The second potential reason is that we employ different attention mechanisms for spatial and temporal encoders. We explored the effectiveness of the attention used by constructing ablation experiments and visualizations of attention. In spatial encoder, SEA mechanism is able to differentiate the importance of each sub-region and augment the important region features, which helps Vahagn to effectively perceive spatial information such as grasping position and target posture. Meanwhile, in temporal encoder, the SA mechanism is implied, it could extracts the relevance of individual frames in the sequences.

## 5 Conclusion

In this paper, we propose a new method of visual-haptic feature extraction based on attention mechanism for slip detection. Comparative experiments are conducted on the slip dataset with traditional CNN-LSTM method, as well as VTFSA and TimeSformer, which also are based on attention mechanism.

The experiment results indicate that the Vahagn performs better on the slip detection task, achieving the accuracy of 93.59%. Ablation experiments on spatial-temporal features demonstrate the important role of the encoders in extracting temporal and spatial information. Additionally, ablation experiments on gate units illustrate that the capabilities of balance mechanisms and causal inferences processes, which are investigated in human perception, can also be useful in the field of robotic perception. We then conduct the comparison experiments on the attention used in encoders. The results illustrate that SEA is an appropriate method for identifying critical regions for spatial encoder, whereas SA is more effective for determining criticality between frames for temporal encoder. Meanwhile, the visualization of the attention shows that the Vahagn exhibits better interpretability and predictive performance than traditional models.

The Vahagn proposed in this paper has made notable advancements in joint visual-tactile perception. In the slide detection task with visual and haptic image sequences as input, we integrate the attributes of temporal and spatial dimensions, and combine SEA and SA to construct independent encoders for feature extraction. In the fusion stage, we employ a two-step fusion method and introduce gate units to regulate the relative importance of the different information. In the future, we will continue to delve into the fusion method of visual and haptic information, with the objective of enhancing the comprehensive perceptual capability of robots.

## Data Availability

Publicly available datasets were analyzed in this study. This data can be found at: https://github.com/wkoa/slip_detection.
